# Supplementary material for: Feasibility and Effects of Implementing Multimodal Prehabilitation Before Cytoreductive Surgery in Patients with Ovarian Cancer: The Gynofit Multicenter Study
Source: Cancers (Basel). 2025 Apr 22;17(9):1393. doi: 10.3390/cancers17091393 (PMC12070995; doi:10.3390/cancers17091393)
Supplement: Supplementary file 1 [file cancers-17-01393-s001.zip › cancers-3524969-supplementary.pdf]

Supplementary materials

Table S1. Patient, clinical and surgical characteristics of the Amphia and Albert Schweitzer cohort. Abbreviations: BMI = body mass index, ASA = American Society of Anesthesiologists, FIGO = International Federation of Gynecology and Obstetrics, HIPEC = hyperthermic intraperitoneal chemotherapy, PCI = peritoneal cancer index.

	**Amphia cohort**					**Albert Schweitzer cohort**		
	**Prehabilitation**	**Controls**		**Non-prehabilitation**		**Prehabilitation**	**Non-prehabilitation**	
Characteristics	**n = 19**	**n = 15**	**p -value**	**n = 9**	**p-value**	**n = 13**	**n = 5**	**p-value**
**Age** (years) *median (IQR)*	71 (55-75)	66 (60-73)	0.40	57 (45-71)	0.15	70.0 (64.5-74.0)	58.0 (49.5-70.0)	0.11
**Body mass index** (kg/m²) *median (IQR)*	24.8 (23.1-27.4)	26.9 (23.0-31.0)	0.73	23.5 (20.7-31.0)	0.40	23.6 (21.4-28.2)	23.8 (22.1-28.7)	0.44
**Comorbidity**	*n = 8 (42%)*	*n = 8 (53%)*	0.73	*n = 3 (30%)*	1.00	*n = 8 (62%)*	*n = 3 (60%)*	0.62
Cardiovascular	5 (26%)	6 (40%)	0.48	2 (22%)	1.00	7 (54%)	3 (60%)	1.00
Pulmonary	2 (11%)	1 (7%)	1.00	-	1.00	1 (8%)	2 (40%)	0.17
Diabetes	-	1 (7%)	0.44	-	-	-	-	-
Other	5 (26%)	3 (20%)	1.00	1 (11%)	0.63	3 (23%)	-	0.35
Charlson Comorbidity Index *mean (±SD)*	7.7 (2.6)	7.7 (2.0)	0.98	6.7 (1.6)	0.35	7.9 (1.7)	7.8 (1.6)	0.89
**ASA**								
I	-	2 (13%)		1 (11%)				
II	11 (58%)	6 (40%)		7 (78%)		5 (38%)	3 (60%)	
III	7 (37%)	7 (47%)	0.26	1 (11%)	0.17	5 (38%)	1 (20%)	0.43
IV	-	-		-		2 (15%)	-	
Missing	1 (5%)	-		-		n = 1	n = 1	
**Histology**, n (%)								
Low-grade serous carcinoma	-	2 (13%)		2 (22%)		-	2 (40%)	
High-grade serous carcinoma	15 (79%)	12 (80%)		6 (67%)		12 (92%)	2 (40%)	
Endometrioid carcinoma	3 (16%)	-		-		-	-	
Clear cell carcinoma	-	1 (7%)	0.11	-	0.06	-	-	**0.02**
Squamous cell carcinoma	-	-		1 (11%)		-	-	
Mucinous carcinoma	-	-		-		-	1 (20%)	
Unclear or unknown	1 (5%)	-		-		1 (6%)	-	
**FIGO stage**, n (%)								
II	3 (16%)	2 (13%)		-		1 (8%)	-	
III	7 (37%)	5 (33%)	1.00	5 (56%)	0.53	2 (15%)	-	0.68
IV	9 (47%)	8 (53%)		4 (44%)		10 (77%)	5 (100%)	
**Recurrent disease**						-	-	
First recurrence	n = 1	n = 1	-	n = 1	-	-	-	-
Second recurrence	n = 1	-		-		-	-	
**Cytoreductive surgery**								
Yes	18 (95%)	15 (100%)	1.00	9 (100%)	1.00	7 (54%)	2 (40%)	1.00
No	1 (5%)	-		-		6 (46%)	3 (60%)	
**Preoperative values**								
Albumin (g/L) *median (IQR)*	36 (33-37)	37 (36-38)	0.10	36 (34-36)	0.66	36 (29.8-38.5)	30.5 (29-32)	0.32
Missing	n = 1	n = 4		n = 3		n = 3	n = 1	
Hemoglobin (mmol/L) *mean (±SD)*	7.0 (0.8)	7.0 (1.0)	0.92	7.7 (1.1)	0.07	7.1 (0.7)	6.5 (2.1)	0.28
Missing	-	-		-		-	n = 1	
CA125 (kU/L) *median (IQR)*	66 (23-127)	45 (27-93)	0.63	67 (36-182)	0.60	54 (17-537)	770 (361-770)	0.29
Missing	n = 1	-		-		n = 1	n = 1	
**Neoadjuvant treatment**								
None	3 (17%)	5 (33%)		4 (44%)		-	-	
3 cycles of chemotherapy	12 (67%)	7 (47%)	0.51	4 (44%)	0.39	6 (86%)	1 (50%)	1.00
4 cycles of chemotherapy	-	-				1 (14%)	1 (50%)	
6 cycles of chemotherapy	3 (17%)	3 (20%)		1 (11%)		-	-	
**Neoadjuvant chemotherapy**								
Dose reduction	n.a.	n.a.	-	n.a.	-	1 (14%)	-	1.00
Deferral	n.a.	n.a.		n.a.		1 (14%)	-	1.00
**Type of surgery**								
Primary cytoreductive surgery	3 (17%)	5 (33%)		4 (44%)		-	-	
Interval cytoreductive surgery	11 (61%)	9 (60%)		4 (44%)	0.18	6 (86%)	2 (100%)	
Interval cytoreductive surgery + HIPEC	3 (17%)	-	0.24	-		1 (14%)	-	1.00
Secondary cytoreductive surgery	-	1 (7%)		1 (11%)		-	-	
Tertiary cytoreductive surgery	1 (6%)	-		-		-	-	
**Surgical complexity score**								
Low	5 (28%)	4 (27%)		2 (22%)		6 (86%)	2 (67%)	
Intermediate	9 (50%)	7 (47%)	1.00	5 (56%)	1.00	1 (14%)	-	1.00
High	4 (22%)	4 (27%)		2 (22%)		-	-	
**PCI** *mean (±SD)*	11.9 (6.5)	11.0 (6.6)	0.74	9.3 (5.0)	0.37	11.7 (5.7)	-	-
Missing	n = 4	n = 3		n = 3		n = 4	n = 2	
**Duration of surgery** (min) *mean (±SD)*	234 (74)	193 (45)	0.07	185 (64)	0.14	254 (87)	257 (96)	0.96
**Intraoperative blood loss** (ml) *median (IQR)*	775 (400-1.200)	300 (500-900)	0.37	550 (250-750)	0.24	1049 (471)	1000 (283)	0.90
**Need for blood transfusion during surgery**								
Yes	4 (22%)	2 (13%)	0.76	1 (11%)	0.64	3 (43%)	-	0.50
No	14 (78%)	13 (87%)		8 (89%)		4 (57%)	2 (100%)	
**Residual disease after surgery**								
Yes	5 (28%)	1 (7%)	0.12	-	0.14	1 (14%)	-	1.00
No	13 (72%)	14 (93%)		9 (100%)		6 (86%)	2 (100%)	
